# Association between the triglyceride-glucose (TyG) index and increased blood pressure in normotensive subjects: a population-based study

**DOI:** 10.1186/s13098-022-00927-5

**Published:** 2022-10-29

**Authors:** Dong-Hwa Lee, Jong Eun Park, So Young Kim, Hyun Jeong Jeon, Jong-Hyock Park

**Affiliations:** 1 Department of Internal Medicine, Chungbuk National University College of Medicine and Chungbuk National University Hospital, Cheongju, Republic of Korea; 2 Institute of Health & Science Convergence, Chungbuk National University, Cheongju, Republic of Korea; 3 Department of Public Health and Preventive Medicine, Chungbuk National University Hospital, Cheongju, Korea; 4 Department of Medicine, College of Medicine, Chungbuk National University, 1 Chungdae-ro, Seowon-gu, Cheongju, 28644 Chungbuk Republic of Korea

**Keywords:** Blood pressure, Hypertension, Triglyceride-glucose index, Korea National Health and Nutrition Examination Survey

## Abstract

**Background:**

Insulin resistance (IR) is an important contributor to the development of hypertension (HTN), and the triglyceride-glucose (TyG) index has been proposed as a simple, reliable marker of IR. This study investigated the association between the TyG index and blood pressure (BP) elevation in a large general population.

**Methods:**

The study enrolled 15,721 adults with no history of cardiometabolic diseases from the 2016–2019 Korea National Health and Nutrition Examination Survey. Participants were classified into quartiles based on the TyG index and BP was categorized as normal BP, elevated BP, pre-HTN, and HTN. The associations of the TyG index with BP categories were assessed using multivariate multinomial logistic regression models with normal BP as the reference group.

**Results:**

The mean systolic/diastolic BP and prevalence of HTN increased with the TyG index (*P* for trend < 0.001). The continuous TyG index had a strong dose-response relationship with increased odds of elevated BP, pre-HTN, and HTN. Compared with the lowest TyG index quartile, the highest TyG index quartile was significantly associated with higher odds of having elevated BP (odds ratio [OR], 1.52; 95% confidence interval [CI], 1.24–1.87; *P* for trend < 0.001), pre-HTN (OR, 2.22; 95% CI, 1.95–2.53; *P* for trend < 0.001), and HTN (OR, 4.24; 95% CI, 3.49–5.16; *P* for trend < 0.001).

**Conclusion:**

We found that a higher TyG index was positively associated with the risk of increased BP in normal healthy individuals. This study suggests that the TyG index might serve as a potential predictor of HTN. However, further studies with larger sample sizes and various target populations in longitudinal designs are needed.

**Supplementary Information:**

The online version contains supplementary material available at 10.1186/s13098-022-00927-5.

## Background

Hypertension (HTN) is an important public health problem because of its increasing prevalence and association with complications such as cardiovascular disease (CVD) [1]. According to the Korea Hypertension Fact Sheet 2020, the number of people with HTN increased from 3.0 million in 2002 to 9.7 million in 2018 [2]. Considering the impact of HTN on morbidity and mortality, there is a need for early predictors of incident HTN, especially in healthy individuals.

Insulin resistance (IR) is an important contributor to the development of HTN [3, 4]. Recently, the triglyceride-glucose (TyG) index, which is calculated using fasting triglycerides and plasma glucose (FPG), was suggested to be a reliable surrogate marker for IR [5, 6]. Several studies demonstrated that the TyG index is correlated with the homeostasis model assessment of insulin resistance (HOMA-IR) and better predicted IR than the HOMA-IR in some studies [5, 7–9]. Therefore, many studies have evaluated the TyG index as a predictor of diseases associated with IR, such as type 2 diabetes, metabolic syndrome, non-alcoholic fatty liver disease, and CVD [10–13]. Studies have demonstrated an association between the TyG index and HTN [14–16], albeit in different study populations.

No study has examined whether the TyG index can predict the risk of HTN in apparently healthy individuals. Therefore, this study evaluated the association between the quartiles of the TyG index and risk of HTN using data from the Korea National Health and Nutrition Examination Survey (KNHANES). Moreover, potential influences of obesity or insulin resistance on the association were also analyzed.

## Methods

### Data sources and study population

The study data were from the 7th (2016–2018) and 8th (2019) KNHANES conducted by the Korea Disease Control and Prevention Agency (KDCA). The KNHANES is a nationally representative cross-sectional survey that assesses the health and nutritional status of Koreans and monitors trends in health risk factors and the prevalence of major chronic diseases [17].

**Fig. 1:**
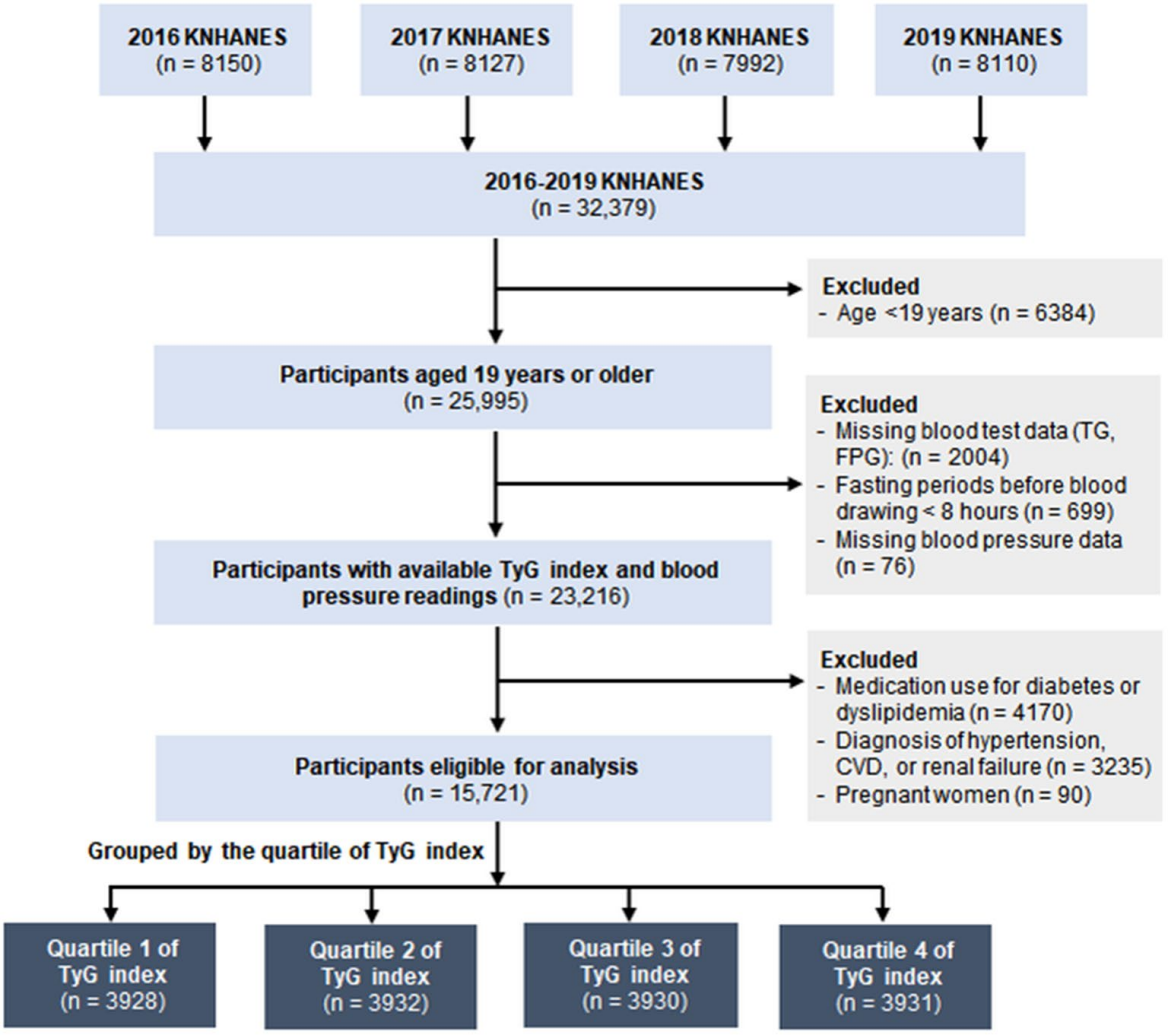
Flowchart of subject selection in the study

Of 25,995 adults aged 19 and over in the 2016–2019 KNHANES database, blood glucose and triglycerides levels measured after a fast of at least 8 h were available for 23,292 participants (Fig. 1). We also excluded participants whose blood pressure (BP) was not recorded (n = 76); who were taking diabetes medications (oral antihyperglycemic agents and insulins) or antihyperlipidemic agents (n = 4170); who self-reported that they had been previously diagnosed with HTN, myocardial infarction, angina, stroke, or renal failure by a physician (n = 3235); or who were pregnant women (n = 90). Finally, 15,721 participants with complete information were included in this analysis and categorized according to the quartiles of the TyG index.

### Definitions of TyG index and BP categories

Blood samples of individuals for biochemical tests were collected after at least 8 h of overnight fasting at a mobile examination center according to examination protocols. FPG was measured by a hexokinase UV method and triglycerides were measured by enzymatic methods. The TyG index was calculated as ln[fasting triglycerides (mg/dL) × fasting glucose (mg/dL) / 2], and classified into quartiles to investigate the association between the TyG index and BP.

The study outcomes were the blood pressure status of participants who were not currently receiving medication for diabetes and hyperlipidemia, and had not previously been diagnosed with hypertension or cardiovascular disease. Systolic (SBP) and diastolic (DBP) blood pressure were measured three times by trained nurses with a mercury sphygmomanometer (Baumanometer Wall Unit 33 [0850], W.A. Baum, NY, USA), at 5-minute intervals in a sitting position after a 5-minute rest. The average of the second and third measurements was used in the analysis. BP categories are based on the classification recommended by the Korean Society of Hypertension [18], and were classified as normal (SBP < 120 mmHg and DBP < 80 mmHg), elevated (SBP 120–129 mmHg and DBP < 80 mmHg), pre-HTN (SBP 130–139 mmHg or DBP 80–89 mmHg), and HTN (SBP ≥ 140 mmHg or DBP ≥ 90 mmHg).

### Laboratory assessment and anthropometric measurements

All laboratory and anthropometric measurements were performed as part of a health checkup according to standard operational procedures. Hemoglobin A1c (HbA1c) was measured by high-performance liquid chromatography (HPLC); total cholesterol was measured by enzymatic methods; high-density lipoprotein (HDL) cholesterol was measured using a homogeneous enzymatic colorimetric method; low-density lipoprotein *(*LDL) cholesterol was measured directly using a homogeneous enzymatic colorimetric method (for the participants with triglycerides > 200) or calculated using the Friedewald equation as total cholesterol – HDL-cholesterol – (triglycerides/5) (mg/dL); aspartate (AST) and alanine (ALT) aminotransferase levels were measured using the International Federation of Clinical Chemistry and Laboratory Medicine UV method without pyridoxal-5-phosphate (P5P). During the observation period, insulin testing was performed only in 2019 and was measured using an electrochemiluminescence immunoassay (ECLIA). Therefore, the homeostasis model assessment of insulin resistance (HOMA-IR) was also calculated only in 2019 using the equation: fasting glucose (mg/dL) × fasting insulin (uIU/mL)/405 [19].

All anthropometric measurements were made by trained examiners using standardized methods. Height and weight were measured with light clothes and without shoes. Body mass index (BMI), calculated by dividing weight by height squared (kg/m^2^), was divided into four categories according to Asian-Pacific guidelines: < 18.5, 18.5–22.9, 23.0–24.9, and ≥ 25.0 kg/m^2^ [20]. Waist circumference was measured to the nearest 0.1 cm at the midpoint between the lower border of the rib cage and the iliac crest while the participants were wearing light clothes. Abdominal obesity was defined as a waist circumference ≥ 90 cm in men and ≥ 85 cm in women in accordance with the definition of the Korean Society for the Study of Obesity (KSSO) [21].

### Assessing sociodemographic and lifestyle variables

The participants’ sociodemographic characteristics included age, sex, marital status, education level, and household income (quartiles). Participants were also asked about their smoking status (non-, former, or current-smoker) and frequency of heavy episodic drinking (does not drink, never in past year, once a month or less, once a week, and almost daily). Heavy episodic drinking was defined as consuming seven or more drinks on a single occasion for men, or five or more drinks on a single occasion for women. We also assessed the family history of hypertension in the parents.

### Statistical analyses

Differences in general characteristics between quartile groups of the TyG index were assessed using the chi-square test for categorical variables and analysis of variance (ANOVA) with Dunnett’s post-hoc analysis for continuous variables. Average SBP and DBP according to the quartile of the TyG index were compared using a general linear model (GLM) after adjusting for survey year, age, and sex. After checking for multicollinearity among the independent variables, multiple linear regression analysis with stepwise selection was performed to identify the combination of risk factors that best explained the variance in BP. We chose the list of correlates considered for introduction in this analysis based on the literature and whether they had a P value < 0.05 in the unadjusted analysis. The following 16 variables were considered in the stepwise regression analysis: age, sex, marital status, education level, household income, smoking, alcohol drinking, BMI, FPG, triglycerides, HDL-cholesterol, LDL-cholesterol, AST, ALT, and TyG index. In the subgroup analysis of 2019 data, fasting insulin levels and insulin resistance status were also considered.

The associations between the TyG index and prevalence of elevated BP, pre-HTN, and HTN were assessed using multinomial logistic regression with normal BP as the reference group. Lipid profiles were not included as covariates in the final logistic regression model to avoid over-adjustment. Participants were assigned the median value for each category to test for trends across each quartile of the TyG index, and this variable was treated as a continuous term in the model. To evaluate the potential for effect modification of TyG index quartiles and BP categories (elevated BP, pre-HTN, or HTN), we stratified the analyses by age (< 50 and ≥ 50 years), sex, BMI (< 25 and ≥ 25 kg/m^2^), and insulin resistance status (HOMA-IR < 2.5 and ≥ 2.5). A cross-product interaction term was included in the multinomial logistic regression model and the statistical significance of the interactions was assessed using the Wald test. All statistical analyses were performed using SAS ver. 9.4 (SAS Institute, Cary, NC, USA).

## Results

### Baseline characteristics

The analysis included 15,721 eligible subjects (6820 males, 8901 females). Table 1 presents the baseline characteristics of the subjects according to the TyG index quartiles. Compared with participants in the lowest TyG quartile, individuals in higher quartiles tend to be older, male, more prone to obesity, less educated, earn less, and have more bad habits, such as current smoking and heavy alcohol consumption (all *P* < 0.001). Furthermore, laboratory findings, including the glycemic markers, fasting insulin, lipid parameters, and liver function, also differed significantly among the TyG index quartiles (all *P* < 0.001). Dunnett’s *post-hoc* test revealed that subjects in the second to fourth quartiles of the TyG index had significantly higher laboratory findings than those in the lowest quartile.

**Table 1:** Comparison of characteristics according to the quartile of TyG index (n = 15,721)

	Quartile of TyG index				*P* value^a^
	Quartile 1 (n = 3928)	Quartile 2 (n = 3932)	Quartile 3 (n = 3930)	Quartile 4 (n = 3931)	
Range of TyG index	6.659–8.055	8.056–8.465	8.466–8.919	8.920–12.208	
Survey year					0.353
2016	947 (24.1)	930 (23.7)	970 (24.7)	1014 (25.8)	
2017	982 (25.0)	1000 (25.4)	1016 (25.9)	946 (24.1)	
2018	979 (24.9)	1022 (26.0)	971 (24.7)	998 (25.4)	
2019	1020 (26.0)	980 (24.9)	973 (24.8)	973 (24.8)	
Age (years)	40.0 ± 14.6	44.4 ± 15.1*	47.5 ± 14.9*	48.1 ± 13.8*	< 0.001
19–29	1120 (28.5)	722 (18.4)	512 (13.0)	349 (8.9)	< 0.001
30–39	981 (25.0)	900 (22.9)	759 (19.3)	805 (20.5)	
40–49	889 (22.6)	901 (22.9)	932 (23.7)	1066 (27.1)	
50–64	659 (16.8)	975 (24.8)	1155 (29.4)	1200 (30.5)	
≥ 65	279 (7.1)	434 (11.0)	572 (14.6)	511 (13.0)	
Sex					< 0.001
Male	991 (25.2)	1427 (36.3)	1906 (48.5)	2496 (63.5)	
Female	2937 (74.8)	2505 (63.7)	2024 (51.5)	1435 (36.5)	
Marital status					< 0.001
Married	2381 (60.6)	2657 (67.6)	2808 (71.5)	2860 (72.8)	
Widowed, separated, or divorced	261 (6.7)	326 (8.3)	369 (9.4)	390 (9.9)	
Single	1285 (32.7)	948 (24.1)	752 (19.1)	679 (17.3)	
Education level					< 0.001
Elementary school graduate or less	252 (6.4)	346 (8.8)	428 (10.9)	458 (11.7)	
Middle school graduate	190 (4.8)	248 (6.3)	315 (8.0)	302 (7.7)	
High school graduate	1334 (34.0)	1378 (35.1)	1314 (33.4)	1326 (33.7)	
College graduate or more	2007 (51.1)	1787 (45.5)	1704 (43.4)	1627 (41.4)	
Unknown	145 (3.7)	173 (4.4)	169 (4.3)	218 (5.6)	
Household income quartiles					< 0.001
Quartile 1 (poorest)	396 (10.1)	473 (12.1)	486 (12.4)	544 (13.9)	
Quartile 2	907 (23.2)	912 (23.3)	949 (24.2)	957 (24.4)	
Quartile 3	1148 (29.3)	1198 (30.6)	1182 (30.2)	1197 (30.5)	
Quartile 4 (richest)	1463 (37.4)	1339 (34.1)	1298 (33.2)	1223 (31.2)	
Smoking status					< 0.001
Non-smoker	3083 (79.0)	2704 (69.5)	2352 (60.5)	1743 (44.8)	
Former smoker	423 (10.8)	587 (15.1)	731 (18.8)	908 (23.4)	
Current smoker	397 (10.2)	600 (15.4)	807 (20.8)	1237 (31.8)	
Frequency of heavy episodic drinking					< 0.001
Dose not drink	790 (20.2)	888 (22.8)	880 (22.6)	730 (18.8)	
Never in past year	1132 (29.0)	1124 (28.9)	947 (24.3)	807 (20.8)	
Once a month or less	1470 (37.7)	1272 (32.7)	1245 (32.0)	1161 (29.9)	
Once a week	414 (10.6)	470 (12.1)	593 (15.2)	826 (21.2)	
Almost daily	98 (2.5)	139 (3.6)	227 (5.8)	364 (9.4)	
Family history of hypertension					< 0.001
No	2358 (60.0)	2363 (60.1)	2386 (60.7)	2313 (58.8)	
Yes	1471 (37.5)	1432 (36.4)	1387 (35.3)	1425 (36.3)	
Unknown	99 (2.5)	137 (3.5)	157 (4)	193 (4.9)	
BMI (kg/m^2^)	21.8 ± 2.9	22.8 ± 3.2*	24.0 ± 3.4*	25.4 ± 3.5*	< 0.001
Underweight (< 18.5)	367 (9.4)	250 (6.4)	107 (2.7)	36 (0.9)	< 0.001
Normal (18.5–22.9)	2415 (61.6)	2020 (51.5)	1512 (38.5)	932 (23.8)	
Overweight (23.0–24.9)	641 (16.4)	819 (20.9)	994 (25.3)	979 (24.9)	
Obesity (≥ 25.0)	497 (12.7)	836 (21.3)	1311 (33.4)	1978 (50.4)	
Waist circumference (cm)	74.7 ± 8.3	78.4 ± 9.0*	82.6 ± 9.4*	87.3 ± 9.1*	< 0.001
Normal (M: < 90, F: < 85)	3620 (92.3)	3307 (84.2)	2839 (72.4)	2234 (57.0)	< 0.001
Abdominal obesity (M: ≥ 90, F: ≥ 85)	304 (7.8)	620 (15.8)	1082 (27.6)	1688 (43.0)	
FPG (mg/dL)	88.9 ± 7.6	92.8 ± 8.1*	96.2 ± 10.8*	106.3 ± 28.0*	< 0.001
HbA1c (%)	5.3 ± 0.3	5.4 ± 0.3*	5.5 ± 0.4*	5.8 ± 0.9*	< 0.001
Fasting insulin (uIU/mL)^a^	5.8 ± 3.4	7.2 ± 4.7*	8.4 ± 5.4*	11.6 ± 11.0*	< 0.001
HOMA-IR^a^	1.3 ± 0.8	1.7 ± 1.1*	2.0 ± 1.4*	3.1 ± 3.6*	< 0.001
Non-insulin resistant (< 2.5)	959 (94.1)	840 (85.7)	741 (76.2)	515 (52.9)	< 0.001
Insulin resistant (≥ 2.5)	60 (5.9)	140 (14.3)	232 (23.8)	458 (47.1)	
Triglycerides (mg/dL)	53.9 ± 11.3	84.9 ± 11.6*	124.5 ± 19.3*	249.7 ± 147.1*	< 0.001
Total cholesterol (mg/dL)	182.6 ± 31.3	193.3 ± 32.1*	202.8 ± 33.5*	214.4 ± 37.1*	< 0.001
HDL-cholesterol (mg/dL)	60.5 ± 12.3	55.5 ± 11.8*	50.8 ± 11.2*	44.3 ± 9.9*	< 0.001
LDL-cholesterol (mg/dL)	111.3 ± 27.5	120.8 ± 29.4*	127.2 ± 31.6*	126.8 ± 33.8*	< 0.001
AST (IU/L)	19.9 ± 9.2	21.0 ± 9.6*	22.3 ± 9.9*	26.7 ± 19.5*	< 0.001
ALT (IU/L)	15.5 ± 11.0	18.0 ± 13.2*	22.2 ± 18.3*	31.1 ± 26.1*	< 0.001

Data are presented as n (%) or mean ± standard deviation

*TyG* triglyceride and glucose, *BMI* body mass index, *FPG* fasting plasma glucose, *HbA1c* hemoglobin A1c, *HOMA-IR* homeostatic model assessment for insulin resistance, *HDL* high-density lipoprotein, *LDL* low-density lipoprotein, *AST* aspartate transaminase, *ALT* alanine aminotransferase

*Represents values significantly different (*P* < 0.05) from control group (the lowest TyG quartile) by the one-way ANOVA followed by the Dunnett's *post-hoc analysis*

^a^Fasting insulin and HOMA-IR were analyzed only for 3945 subjects who participated in the 2019 KNHANES

### Distribution of BP and prevalence of elevated BP, pre-HTN, and HTN according to TyG index quartiles

**Fig. 2:**
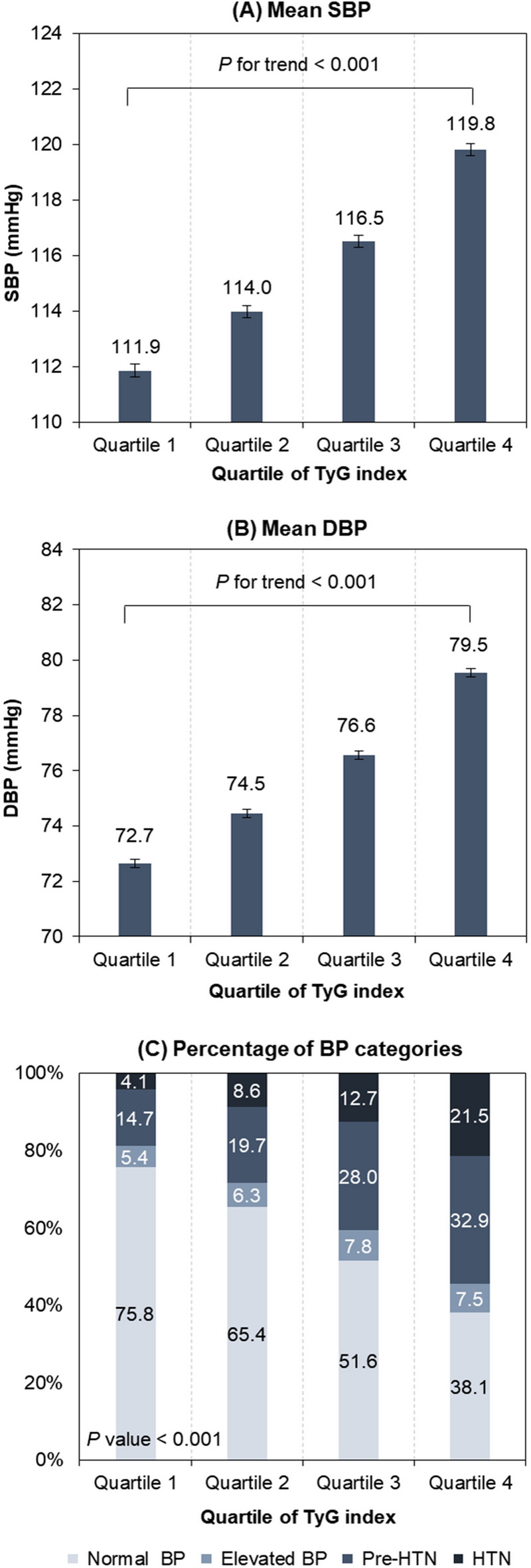
Distribution of blood pressure according to the quartile of the TyG index. Mean and standard error of **A** systolic blood pressure (SBP) and **B** diastolic blood pressure (DBP) adjusted for survey year, age, and sex. **C** Percentage distribution of blood pressure categories

The distribution of BP according to the quartile of TyG index is shown in Fig. 2A and B. SBP and DBP differed significantly among the TyG index quartiles, and had the lowest values in the lowest TyG index quartile. Both SBP and DBP increased with the TyG index (all *P* for trend < 0.001).

Figure 2C shows the percentage distribution of BP categories according to the TyG index quartile. The percentage of participants defined as HTN was highest in the highest TyG quartile and lowest in the lowest TyG quartile (21.5% vs. 4.1%, *P* < 0.001 among groups). The percentage increased with the TyG index quartile. Similar trends were observed for the participants classified as pre-HTN (32.9% vs. 14.7%).

Similarly, in the subgroup analysis based on the insulin resistance status (Additional file 1: Figure S1), as the TyG index increased, the prevalence of pre-HTN and HTN increased in both the non-insulin and the insulin resistant groups (all *P* < 0.001).

### Independent correlates of blood pressure variability

Table 2 shows the results of multiple regression analysis with stepwise variable selection to identify the aggregate combination of correlates making the greatest contribution to BP changes. Stepwise linear regression revealed that among the 16 entered variables, the most important correlates of SBP were the combination of age, sex, marital status, education level, household income, smoking status, frequency of heavy episodic drinking, family history of hypertension, BMI, FPG, HDL-cholesterol, AST, and TyG index, accounting for 26.43% of the SBP variance in this population (F = 429.47, *P* < 0.001). Age, sex, household income, smoking status, frequency of heavy episodic drinking, family history of hypertension, BMI, HDL-cholesterol, LDL-cholesterol, ALT, and the TyG index independently affected DBP, explaining 20.80% of the variance in DBP (F = 371.14, *P* < 0.001). In particular, higher age, BMI and TyG index, and male sex had greater correlations with BP variability. In the subgroup analysis of 2019 data, which also considered fasting insulin and HOMA-IR, similar results were observed.

**Table 2:** Results of stepwise multiple regression analysis for predictors of blood pressure variability

	SBP			DBP		
	Standardized coefficients β	*t*	*P* value	Standardized coefficients β	*t*	*P* value
All subjects (n = 15,721)						
Constant		20.63	< 0.001		15.42	< 0.001
Age	0.341	40.04	< 0.001	0.089	11.13	< 0.001
Sex (male)	0.148	17.96	< 0.001	0.148	17.25	< 0.001
Marital status (married)	-0.089	− 11.55	< 0.001	Not entered		
Education level (college graduate or more)	-0.078	− 10.33	< 0.001	Not entered		
Household income quartiles	-0.033	− 4.46	< 0.001	0.023	3.10	0.002
Smoking status (current smoker)	-0.040	− 5.14	< 0.001	− 0.030	− 3.67	< 0.001
Frequency of heavy episodic drinking	0.063	7.96	< 0.001	0.106	12.75	< 0.001
Family history of hypertension (yes)	0.054	7.76	< 0.001	0.094	13.02	< 0.001
BMI	0.193	25.02	< 0.001	0.198	23.85	< 0.001
FPG	0.036	4.52	< 0.001	Not entered		
Triglycerides	Not entered			Not entered		
HDL-cholesterol	0.099	11.69	< 0.001	0.107	12.23	< 0.001
LDL-cholesterol	Not entered			0.048	6.29	< 0.001
AST	0.028	3.94	< 0.001	Not entered		
ALT	Not entered			0.045	5.57	
TyG index	0.152	16.2	< 0.001	0.220	24.17	< 0.001
Adjusted *R*^*2*^	0.2643 (*F* = 429.47, *P* < 0.001)			0.2080 (*F* = 371.14, *P* < 0.001)		
Subjects in 2019 KNHANES (n = 3945)^a^						
Constant		11.23	< 0.001		9.26	< 0.001
Age	0.376	22.19	< 0.001	0.103	6.35	< 0.001
Sex (male)	0.129	8.15	< 0.001	0.124	7.46	< 0.001
Marital status (married)	-0.079	− 5.15	< 0.001	Not entered		
Education level (college graduate or more)	-0.086	− 5.83	< 0.001	Not entered		
Household income quartiles	Not entered			Not entered		
Smoking status (current smoker)	Not entered			Not entered		
Frequency of heavy episodic drinking	0.054	3.39	< 0.001	0.094	5.66	< 0.001
Family history of hypertension (yes)	0.059	4.15	< 0.001	0.093	6.29	< 0.001
BMI	0.196	12.66	< 0.001	0.194	11.53	< 0.001
FPG	Not entered			Not entered		
Fasting insulin	Not entered			Not entered		
Insulin resistance status (HOMA-IR ≥ 2.5)	Not entered			Not entered		
Triglycerides	Not entered			Not entered		
HDL-cholesterol	0.105	6.10	< 0.001	0.116	6.44	< 0.001
LDL-cholesterol	Not entered			0.039	2.54	0.011
AST (IU/L)	Not entered			Not entered		
ALT (IU/L)	Not entered			0.037	2.32	0.020
TyG index	0.132	7.50	< 0.001	0.201	10.74	
Adjusted *R*^*2*^	0.2440 (*F* = 139.98, *P* < 0.001)			0.1745 (*F* = 92.01, *P* < 0.001)		

The selection of model variables used a “stepwise” option with variable selection criteria: “slentry” = 0.05, “slstay” = 0.1

*SBP* systolic blood pressure, *DBP* diastolic blood pressure, *BMI* body mass index, *FPG* fasting plasma glucose, *HDL* high-density lipoprotein, *LDL* low-density lipoprotein, *AST* aspartate transaminase, *ALT* alanine aminotransferase, *TyG* triglyceride and glucose, *HOMA-IR* homeostatic model assessment for insulin resistance

^a^ Only 3,945 subjects with available data on fasting insulin and HOMA-IR were analyzed

### Association of TyG index with elevated BP, pre-HTN and HTN

We used multinomial logistic regression analysis to evaluate the association of the TyG index with the prevalence of elevated BP, pre-HTN, and HTN (Table 3). The ORs of elevated BP, pre-HTN, and HTN increased with the TyG index quartiles (all *P* for trend < 0.001). Specifically, participants in the highest TyG index quartile had 1.82-fold higher odds of elevated BP (95% CI, 1.50–2.21), 2.95-fold higher odds of pre-HTN (95% CI, 2.62–3.34), and 6.46-fold higher odds of HTN (95% CI, 5.36–7.78) than the lowest TyG index quartile group in the age- and sex-adjusted model. Even after adjusting for conventional risk factors of HTN, such as demographic factors, health behavior (smoking and alcohol drinking), family history of hypertension, and BMI, participants in the highest TyG index quartile were most prominently associated with higher prevalence of elevated BP (OR, 1.52; 95% CI, 1.24–1.87), pre-HTN (OR, 2.22; 95% CI, 1.95–2.53), and HTN (OR, 4.24; 95% CI, 3.49–5.16). We also observed significant dose–response relationships between the continuous TyG index and BP categories.

**Table 3:** Multinomial logistic regression model for the association of TyG index quartiles with elevated blood pressure, pre-hypertension, and hypertension

	Continuous TyG index	Quartile of TyG index				*P* for trend
		Quartile 1	Quartile 2	Quartile 3	Quartile 4	
Elevated BP						
No. of cases	1058	210	247	306	295	
Model 1: OR (95% CI)	1.50 (1.34–1.67)	1.00 (ref.)	1.08 (0.89–1.31)	1.45 (1.20–1.75)	1.82 (1.50–2.21)	< 0.001
Model 2: OR (95% CI)	1.53 (1.36–1.71)	1.00 (ref.)	1.10 (0.90–1.34)	1.49 (1.23–1.80)	1.88 (1.54–2.29)	< 0.001
Model 3: OR (95% CI)	1.34 (1.19–1.51)	1.00 (ref.)	1.05 (0.86–1.28)	1.32 (1.09–1.61)	1.52 (1.24–1.87)	< 0.001
Pre-HTN						
No. of cases	3748	579	774	1100	1295	
Model 1: OR (95% CI)	2.00 (1.87–2.14)	1.00 (ref.)	1.31 (1.16–1.48)	2.05 (1.82–2.32)	2.95 (2.62–3.34)	< 0.001
Model 2: OR (95% CI)	1.97 (1.84–2.11)	1.00 (ref.)	1.31 (1.16–1.49)	2.03 (1.80–2.29)	2.87 (2.54–3.25)	< 0.001
Model 3: OR (95% CI)	1.68 (1.56–1.81)	1.00 (ref.)	1.23 (1.09–1.39)	1.74 (1.54–1.97)	2.22 (1.95–2.53)	< 0.001
HTN						
No. of cases	1842	161	339	497	845	
Model 1: OR (95% CI)	2.94 (2.70–3.20)	1.00 (ref.)	1.93 (1.58–2.35)	3.03 (2.50–3.66)	6.46 (5.36–7.78)	< 0.001
Model 2: OR (95% CI)	2.84 (2.59–3.10)	1.00 (ref.)	1.94 (1.58–2.37)	2.96 (2.44–3.59)	6.12 (5.06–7.39)	< 0.001
Model 3: OR (95% CI)	2.29 (2.09–2.52)	1.00 (ref.)	1.76 (1.44–2.16)	2.38 (1.95–2.90)	4.24 (3.49–5.16)	< 0.001

*TyG* triglyceride and glucose, *BP* blood pressure, *OR* odds ratio, *CI* confidence interval, *HTN* hypertension, *BMI* body mass index

Model 1: adjusted for survey year, age, and sex

Model 2: adjusted for variables in the Model 1 + marital status, education level, household income quartiles, smoking status, frequency of heavy episodic drinking, and family history of hypertension

Model 3: adjusted for variables in the Model 2 + BMI

In the analyses stratified by age, sex, BMI, and insulin resistance status (Table 4), age significantly modified the association between the TyG index and the prevalence of pre-HTN (*P* interaction < 0.001) or HTN (*P* interaction < 0.001), and the associations were more apparent among those who were < 50 years of age. However, the association of the TyG index with BP categories did not differ by sex, except for elevated BP; elevated BP was more prominent in women than in men (*P* interaction = 0.015). There were also no interactions between the TyG index and BMI or insulin resistance status.

**Table 4:** Multinomial logistic regression model for the association of TyG index quartiles with elevated blood pressure, pre-hypertension, and hypertension by age, sex, BMI, and insulin resistance status

		Quartile of TyG index				*P* interaction
		Quartile 1	Quartile 2	Quartile 3	Quartile 4	
By age						
Elevated BP						0.387
Age < 50 years	No. of cases	111	89	100	106	
	OR (95% CI)	1.00 (ref.)	0.87 (0.65–1.17)	1.10 (0.82–1.48)	1.28 (0.94–1.76)	
Age ≥ 50 years	No. of cases	99	158	206	189	
	OR (95% CI)	1.00 (ref.)	1.19 (0.90–1.57)	1.54 (1.17–2.02)	1.66 (1.25–2.21)	
Pre-HTN						< 0.001
Age < 50 years	No. of cases	360	442	575	748	
	OR (95% CI)	1.00 (ref.)	1.30 (1.11–1.53)	1.88 (1.60–2.20)	2.64 (2.23–3.12)	
Age ≥ 50 years	No. of cases	219	332	525	547	
	OR (95% CI)	1.00 (ref.)	1.08 (0.88–1.33)	1.56 (1.28–1.91)	1.76 (1.43–2.16)	
HTN						< 0.001
Age < 50 years	No. of cases	56	121	195	436	
	OR (95% CI)	1.00 (ref.)	2.00 (1.43–2.78)	3.15 (2.29–4.34)	6.68 (4.88–9.13)	
Age ≥ 50 years	No. of cases	105	218	302	409	
	OR (95% CI)	1.00 (ref.)	1.52 (1.17–1.98)	1.95 (1.51–2.52)	2.84 (2.20–3.68)	
By sex						
Elevated BP						0.015
Male	No. of cases	86	113	126	169	
	OR (95% CI)	1.00 (ref.)	0.98 (0.72–1.34)	0.98 (0.72–1.33)	1.32 (0.98–1.79)	
Female	No. of cases	124	134	180	126	
	OR (95% CI)	1.00 (ref.)	1.02 (0.78–1.32)	1.52 (1.17–1.97)	1.56 (1.17–2.08)	
Pre-HTN						
Male	No. of cases	227	368	649	912	0.464
	OR (95% CI)	1.00 (ref.)	1.11 (0.91–1.36)	1.60 (1.32–1.94)	2.03 (1.67–2.46)	
Female	No. of cases	352	406	451	383	
	OR (95% CI)	1.00 (ref.)	1.27 (1.08–1.49)	1.75 (1.48–2.06)	2.29 (1.90–2.75)	
HTN						0.453
Male	No. of cases	63	144	267	576	
	OR (95% CI)	1.00 (ref.)	1.47 (1.06–2.03)	2.11 (1.55–2.86)	3.91 (2.90–5.27)	
Female	No. of cases	98	195	230	269	
	OR (95% CI)	1.00 (ref.)	1.88 (1.45–2.45)	2.40 (1.85–3.12)	3.97 (3.04–5.19)	
By BMI						
Elevated BP						0.475
BMI < 25.0 kg/m^2^	No. of cases	168	182	204	153	
	OR (95% CI)	1.00 (ref.)	1.03 (0.82–1.29)	1.43 (1.14–1.79)	1.56 (1.22–2.00)	
BMI ≥ 25.0 kg/m^2^	No. of cases	41	65	101	141	
	OR (95% CI)	1.00 (ref.)	1.09 (0.71–1.67)	1.14 (0.76–1.70)	1.51 (1.02–2.23)	
Pre-HTN						0.598
BMI < 25.0 kg/m^2^	No. of cases	463	554	676	571	
	OR (95% CI)	1.00 (ref.)	1.23 (1.07–1.41)	1.86 (1.62–2.15)	2.19 (1.88–2.56)	
BMI ≥ 25.0 kg/m^2^	No. of cases	115	217	423	722	
	OR (95% CI)	1.00 (ref.)	1.27 (0.96–1.67)	1.67 (1.30–2.16)	2.45 (1.91–3.14)	
HTN						
BMI < 25.0 kg/m^2^	No. of cases	130	213	271	349	
	OR (95% CI)	1.00 (ref.)	1.50 (1.18–1.90)	2.25 (1.78–2.83)	3.94 (3.12–4.97)	0.698
BMI ≥ 25.0 kg/m^2^	No. of cases	30	124	225	496	
	OR (95% CI)	1.00 (ref.)	2.75 (1.79–4.24)	3.27 (2.16–4.95)	6.26 (4.18–9.36)	
By insulin resistance status^a^						
Elevated BP						0.749
HOMA-IR < 2.5	No. of cases	59	58	64	39	
	OR (95% CI)	1.00 (ref.)	0.92 (0.61–1.37)	1.18 (0.78–1.78)	1.03 (0.64–1.66)	
HOMA-IR ≥ 2.5	No. of cases	7	6	12	39	
	OR (95% CI)	1.00 (ref.)	0.34 (0.09–1.26)	0.41 (0.13–1.32)	0.83 (0.29–2.40)	
Pre-HTN						0.679
HOMA-IR < 2.5	No. of cases	138	189	222	153	
	OR (95% CI)	1.00 (ref.)	1.47 (1.14–1.90)	2.00 (1.53–2.61)	1.86 (1.37–2.52)	
HOMA-IR ≥ 2.5	No. of cases	9	39	68	167	
	OR (95% CI)	1.00 (ref.)	1.91 (0.80–4.54)	1.71 (0.74–3.96)	2.53 (1.12–5.74)	
HTN						0.067
HOMA-IR < 2.5	No. of cases	43	71	85	105	
	OR (95% CI)	1.00 (ref.)	1.49 (0.98–2.27)	1.92 (1.26–2.91)	3.16 (2.06–4.86)	
HOMA-IR ≥ 2.5	No. of cases	6	18	35	84	
	OR (95% CI)	1.00 (ref.)	1.15 (0.39–3.40)	0.98 (0.35–2.78)	1.36 (0.50–3.73)	

Adjusted for survey year, age, sex, marital status, education level, household income quartiles, smoking status, and frequency of heavy episodic drinking, family history of hypertension, and BMI, except for the variable used in each stratified analysis

*TyG* triglyceride and glucose, *BP* blood pressure, *OR* odds ratio, *CI* confidence interval, *HTN* hypertension, *BMI* body mass index, *HOMA-IR* homeostatic model assessment for insulin resistance

^a^Only 3,945 subjects with available data on HOMA-IR were analyzed

## Discussion

In this population-based cross-sectional study, the TyG index was positively associated with the increment in BP. Note that the study participants were apparently healthy individuals with no history of HTN, CVD, or renal failure and were not taking anti-diabetic or antihyperlipidemic medications. Even after adjusting for conventional risk factors, the significant association between the TyG index and BP was maintained. These findings suggest that the TyG index is independently associated with BP and may be useful for identifying and following individuals at risk of HTN.

IR has been implicated in the pathogenesis of diseases related to metabolic syndrome, including HTN, diabetes mellitus, obesity, and CVD [3, 22, 23]. The gold standard for assessing IR is hyperinsulinemic-euglycemic clamp analysis [24]. However, it is difficult to perform in real-world settings because it is time-consuming and labor-intensive. HOMA-IR has been suggested as a simpler method and its results correlated well with those assessed by the clamp analysis [19]. However, it also has limited value because serum insulin is not measured routinely in clinical settings. More recently, the TyG index has been proposed for evaluating IR [5]. In previous studies, the TyG index correlated well with HOMA-IR [5, 7–9]. In our study, although serum insulin was measured in a relatively small number of subjects, HOMA-IR showed a significant correlation with the quartiles of the TyG index. Compared to serum insulin, triglycerides and FPG can be assessed simply and easily; this is more suitable as a mass screening test to predict IR-related diseases, such as in our study.

Although the mechanisms underlying IR in the development of HTN have not been fully elucidated, several have been suggested. Hyperinsulinemia caused by IR may increase the activity of the renin-angiotensin-aldosterone system, which can induce renal sodium retention [25, 26]. It can indirectly cause water-sodium retention and increase vascular activity via angiotensin II, resulting in HTN [27]. IR may also stimulate sympathetic nervous system activity, inducing the secretion of adrenaline and norepinephrine, leading to increased cardiac output and peripheral vascular resistance via vascular smooth muscle cell hypertrophy and endothelial dysfunction [28–30].

Several studies have evaluated the relationship between the TyG index and IR-related disease, especially in HTN. In a 9-year longitudinal study, a higher TyG index was associated with an increased risk of subsequent incident HTN [14]. A large epidemiological study of the temporal relationship between BMI and the TyG index and its impact on the incidence of HTN found that a higher BMI at baseline was significantly associated with a higher TyG index and an increased risk of HTN [31]. In addition, a higher TyG index was significantly associated with a higher BMI at the 2-year follow-up and an increased risk of HTN. These results provide direct evidence for a temporal relationship between BMI and IR. A more recent study demonstrated that an increased TyG index was significantly associated with a higher risk of pre-HTN and HTN [15]. Furthermore, obesity parameters such as the waist-to-hip ratio and percent body fat have additive effects on the HTN risk with the TyG index. Our results also showed a positive correlation between the TyG index and BP and prevalence of HTN in alignment with these previous studies.

This study examined subjects who had low risks for IR. Individuals who had previously been diagnosed with HTN, CVD, or renal failure by a physician or who were receiving medication for diabetes mellitus and dyslipidemia were excluded. Nevertheless, a significant relationship was observed between the TyG index and BP; the mean SBP in the lowest and highest quartiles was 111.9 and 119.8 mmHg, respectively (*P* for trend < 0.001) and mean DBP in the lowest and highest quartiles was 72.7 and 79.5 mmHg (*P* for trend < 0.001). In the subgroup analysis according to BMI, the TyG index was significantly associated with the prevalence of elevated BP, pre-HTN, and HTN, even in the non-obese subjects (BMI < 25 kg/m^2^). Furthermore, the associations between the TyG index and the prevalence of pre-HTN and HTN were more prominent in younger subjects (*P* interaction < 0.001). Overall, these results suggest the potential of the TyG index as a predictor of HTN.

This study has several advantages. It included relatively large number of subjects from a national health survey. We were able to analyze various confounding factors potentially influencing HTN (demographic, lifestyle, and laboratory parameters). In addition, the analysis examined categories of BP and subgroups by subject characteristics. However, our study also has some limitations that need to be considered. First, because of its cross-sectional design, it cannot show a causal relationship between the TyG index and HTN. Second, as KNHANES did not usually include fasting insulin, we could analyze only a small number of subjects and compare the TyG index with the HOMA-IR as an independent risk factor. Third, although we tried to adjust for confounding risk factors, there might be some confounding factors that we did not include.

## Conclusion

In conclusion, a higher TyG index significantly correlated with the risk of increased BP in healthy individuals after adjusting for conventional risk factors of HTN. Considering its simplicity of measurement and good functionality, the TyG index might be a useful marker for identifying patients at risk of HTN. However, further studies are needed to longitudinally investigate the cause-effect relationship between the TyG index and BP.

## Supplementary Information

**Additional file 1: Figure S1.** Percentage distribution of blood pressure according to the quartile of the TyG index in (A) non-insulin resistant and (B) insulin resistant groups. P values were generated by chi-square test.

## Data Availability

The data of the current study are available from the Korea Disease Control and Prevention Agency (https://knhanes.kdca.go.kr/knhanes/main.do) on reasonable request.

## Abbreviations

AST: Aspartate transaminase
ALT: Alanine aminotransferase
BMI: Body mass index
BP: Blood pressure
CI: Confidence interval
CVD: Cardiovascular disease
DBP: Diastolic blood pressure
FPG: Fasting plasma glucose
HDL: High‐density lipoprotein
HOMA-IR: Homeostasis model assessment of insulin resistance
HTN: Hypertension
IR: Insulin resistance
KDCA: Korea Disease Control and Prevention Agency
KNHANES: Korea National Health and Nutrition Examination Survey
LDL: Low-density lipoprotein
OR: Odds ratio
P5P: Pyridoxal-5-phosphate
SBP: Systolic blood pressure
SD: Standard deviation
TyG: Triglyceride-glucose
