# Supplementary material for: Association between the triglyceride-glucose (TyG) index and increased blood pressure in normotensive subjects: a population-based study
Source: Diabetol Metab Syndr. 2022 Oct 29;14:161. doi: 10.1186/s13098-022-00927-5 (PMC9617408; doi:10.1186/s13098-022-00927-5)
Supplement: Supplementary file 1 — Additional file 1: Figure S1. Percentage distribution of blood pressure according to the quartile of the TyG index in (A) non-insulin resistant and (B) insulin resistant groups. P values were generated by chi-square test. [file 13098_2022_927_MOESM1_ESM.docx]

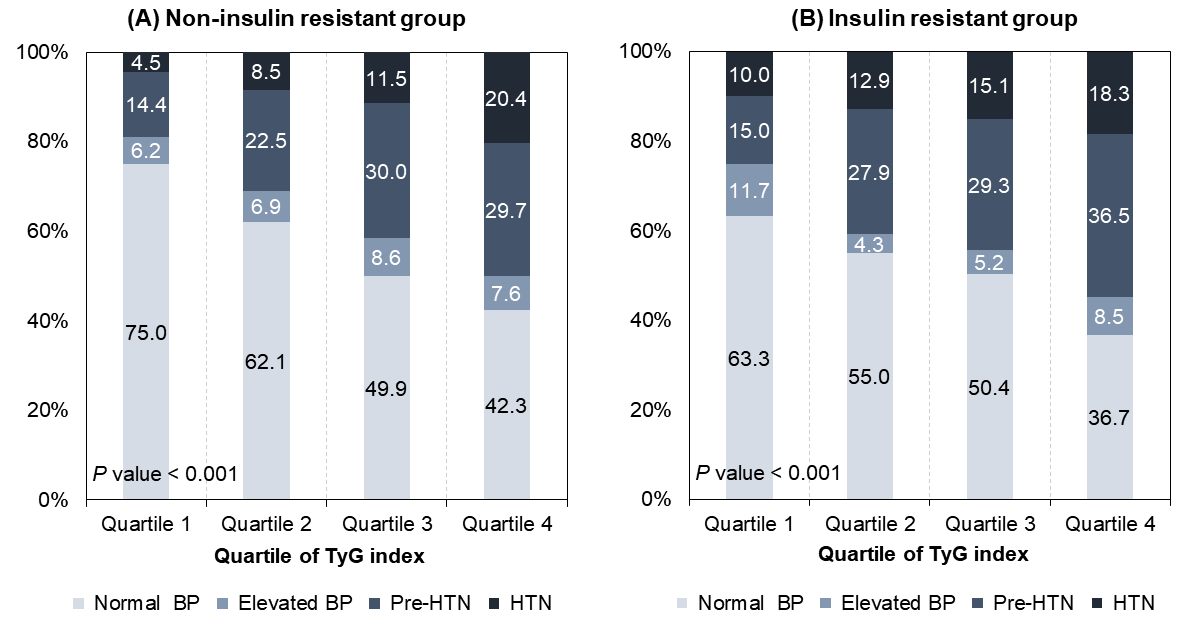


**Supplementary figure 1**. Percentage distribution of blood pressure according to the quartile of the TyG index in (A) non-insulin resistant and (B) insulin resistant groups. *P* values were generated by chi-square test.
